# Investigating the Effect of Autologous Platelet-Rich Plasma on Pain in Patients With Pilonidal Abscess Treated With Surgical Removal of Extensive Tissue

**DOI:** 10.5812/ircmj.6301

**Published:** 2013-11-05

**Authors:** Mostafa Mehrabi Bahar, Mohsen Ali Akbarian, Ali Azadmand

**Affiliations:** 1 Mashhad University of Medical Sciences, Mashhad, IR Iran

**Keywords:** Platelet-Rich Plasma, Wound Healing, Pilonidal Sinus

## Abstract

**Background:**

Pilonidal is a painful disease which occurs mainly in the sacrococcygeal area. In 1833 Herbert Mayo first reported a case of a young woman with a hair-containing sinus in the sacrococcygeal area. In 1880, Hodge suggested the term “pilonidal” from the Latin “pilus” for hair and “nidus” meaning nest.

**Objectives:**

Effect of using autologous platelet-rich plasma (PRP) on pain reduction in patients with pilonidal abscess, subjected to extensive surgical removal tissue, was investigated through a clinical trial.

**Patients and Methods:**

The trial was conducted on two groups from the Center for Surgical Research at Mashhad University of Medical Sciences located at Ghaem hospital in Mashhad city in 2011. Purposive sampling was conducted and the samples were randomly assigned to two groups including test and control. All of the patients referred to the surgical unit of Imam-Reza hospital with acute pilonidal abscess (sacrococcygeal abscess) were considered and randomly assigned to two groups. Patients’ dressing was not removed for two days. To avoid systematic errors (Bias), the resident who checked the wound for the first time was completely unaware of the patient’s treatment plan. Recovery process and wound healing were monitored for the two groups and compared with each other (every other day for 10 days, and then once a week until complete healing). Data analysis was performed using SPSS19 software with Chi-square, independent t, and Mann-Whitney tests.

**Results:**

Based on the obtained results it was found that the pain in the test group was significantly reduced in the first and fourth weeks (P < 0.05), compared to the control group.

**Conclusions:**

Therefore, it has been demonstrated that using PRP can significantly reduce pain in patients subjected to surgical treatment for their pilonidal abscess.

## 1. Background

Pilonidal is a painful disease which occurs mainly in the sacrococcygeal area. In 1833 Herbert Mayo first reported a case of a young woman with a hair-containing sinus in the sacrococcygeal area. In 1880, Hodge suggested the term “pilonidal” from the Latin “pilus” for hair and “nidus” meaning nest (1).

During the World War II, more than 78,924 soldiers were hospitalized with this diagnosis in military hospitals. The disease was caused by frequent reactivation and prolonged sitting during driving the jeeps, and therefore was named as the “jeep disease” (2).

Various treatment methods have been used as surgical treatments for pilonidal sinus including excision with primary closure, excision and leaving the wound open, marsupialization, and different types of flaps (3-7). Three main factors including recurrence rate, wound infections, and cavity or wound healing rate should be considered in choosing among treatment options. Recurrence rate varies in different methods (8). Allen Merch has reported the recurrence rate of 15.4% for primary healing, and 8% for flap in a one-year study (9). Furthermore, Kronborg et al. have reported the recurrence rate of 13% for excision and 25% for excision with primary healing (10).

Local application of autologous platelet concentrate including growth factors is a new method for accelerating wound healing and reducing pain (11). Upon the activation of platelets, the growth factors are released, resulting in acceleration of wound healing process. A number of factors affecting this process include (12, 13): Platelet-Derived Growth Factor (PDGF), Vascular Endothelial Growth Factor (VEGF), Epidermal Growth Factor (EGF), Connective tissue Growth Factor (FGF), and Transforming Growth Factor b (TGF-b1).

In a study conducted by Michail (14), wound healing rate of patients who had received PRP was meaningfully higher than the control group. There are a few studies performed in this field; hence, herein the effect of using PRP on pain reduction in patients with pilonidal abscess subjected to extensive removal surgical tissue was investigated.

## 2. Objectives

The present study was a clinical trial conducted on two groups in the Center for Surgical Research at Mashhad University of Medical Sciences located at Ghaem hospital in Mashhad in 2011.

## 3. Patients and Methods

Sample size and power in each group were calculated with 95% confidence and 37.80 individuals, respectively. Purposive sampling was conducted and the samples were randomly assigned to two groups. All of the patients referred to the surgical unit of Imam-Reza hospital with acute pilonidal abscess (sacrococcygeal abscess) were considered for the study. Consciously written consent was obtained from all the patients. Pain assessment was performed with pain linear visual scale; a vertical line with 10 cm length; in which lack of pain scores zero, and the maximum pain scores 10.

Studied variables included the age, sex, time needed for wound healing, wound depth, postoperative onset of social activities, treatment, smoking, anemia, and body mass index (BMI). At the beginning, patients with pilonidal abscess admitted to the surgical unit were randomly assigned to one of the treatment groups; group A: extensive surgical removal and dressing, group B: extensive surgical removal and application of PRP. After the operation and excision of the abscess, the cavity size was measured accurately (the cavity was filled with 0.09% normal saline solution and then the entire contents of the cavity was drawn with a syringe and was recorded). To gain PRP from the patients of group B, after obtaining their written consent, their blood was drawn as five times the volume of the cavity (max. volume of 250 mL) and was sent to the School of Medicine (New Technology Center). Antibiotics were administered for both groups, and their wounds were washed with normal saline solution twice a day (continued for at least 24 - 48 hours). The PRP preparation procedure took 24 hours and therefore it was performed in the New Technology Center of the Medical School. Drawn blood was immediately transferred to the center, and then centrifuged at 2000 G for 8 minutes to separate RBCs. Next, prepared platelet rich plasma was centrifuged for 15 minutes at 4000 G to sediment the platelets. After decanting the supernatant solution, the final platelet concentrate was obtained. To obtain the Fibrin-glue which facilitates the PRP clotting after injection, plasma was frozen at -20°C, followed by defrosting at 4°C and centrifuging at 2300 G. PRP preparation takes about 30 minutes, while it almost takes 20 hours to obtain fibrinogen. Volume of the obtained solution was about 20 - 25 cc per 100 cc of the blood. The prepared plasma was used immediately and the PRP was injected 24 - 36 hours after the operation. The patient’s dressing was removed, and the wound was investigated carefully for signs of infection and cellulitis. If the wound in patients of group B was ready to apply PRP, the cavity was completely filled with PRP up to the skin surface after washing with normal saline solution, and left for coagulation. Then, the area was covered with Vaseline gas and dressing. If the wound was not ready, washing and using antibiotics were continued to reach the proper condition. Prepared PRP included Ciprofloxacin as well as several factors, which had antibacterial effects.

The wound dressing was not removed for two days. To avoid systematic errors (Bias), the resident who checked the wound for the first time was completely unaware of the patient’s treatment plan. Recovery process and wound healing were monitored for the two groups and compared with each other (every other day for 10 days, and then once a week until complete healing). The cavity volume and possible complications such as infections, bleeding, pain, and etc. were investigated in every visit. Finally, cavity volumes on different days and complications such as infection, full recovery time (complete filling of the wound with natural tissue), postoperative onset of social activities, and etc. were compared for the two groups. Care was continued until full recovery and entire healing of the wound. Data analysis was performed using the SPSS19 software using Chi-square, independent t, and Mann-Whitney tests.

## 4. Results

The average age of the patients was 24.81 ± 3.89 in the test group and 24.70 ± 1.50 in the control group, and there was no significant difference between the members of the groups regarding age (Table 1) and sex (Table 2). 

**Table 1.** Comparison of the Two Groups Regarding Their Characteristics

Variable	Control	Case	P Value
**Age, y ** ^**a**^	24.70 (1.50)	24.81 ± 3.89	0.178
**Smoking**	4	9	0.129
**BMI ** ^**a, b**^	23.864 (1.279)	27.1 ± 1.361	0.000
**Infection rate**	1	4	0.005
**Return to work** ^**a**^	28.189 (1.941)	18.918 ± 1.341	0.000
**Wound healing time ** ^**a**^	34.891 (4.520)	25.513 ± 6.167	0.128
**Wound depth ** ^**a**^	27.513 (7.533)	34.108 ± 12.950	0.810

^a^ Mean (SD).

^b^ BMI, body mass index.

**Table 2.** Comparison of the Two Groups Regarding Sex

Variable	Control	Case	P Value
**Male**	22	20	0.500
**Female**	15	17	0.725

**Figure 1.**
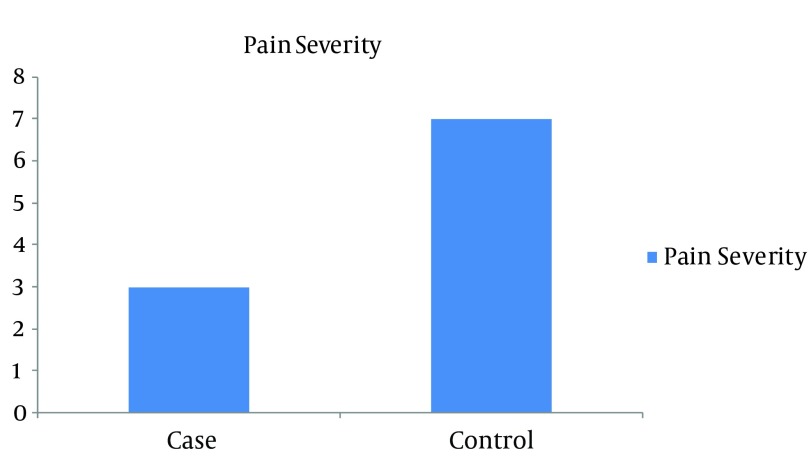
Comparison of Pain Between the Two Groups for the First Week.

**Figure 2.**
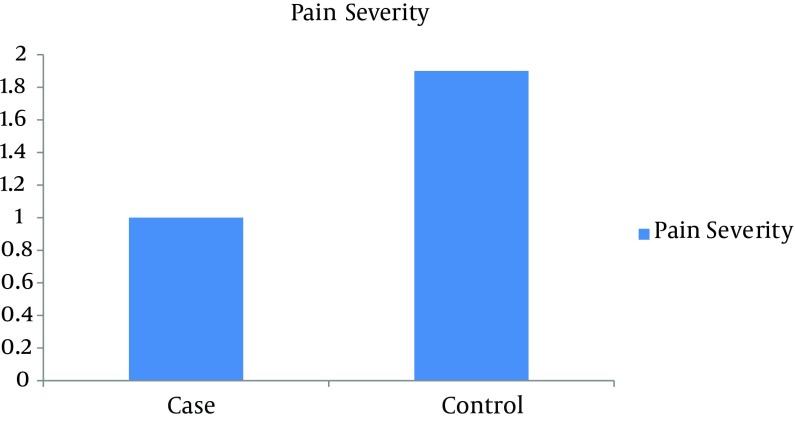
Comparison of Pain Between the Two Groups for the Fourth Week.

During the first week, pain was 3.081 ± 0.4932 for the test group, and 7.054 ± 0.328 for the control group and their meaningful difference can be seen in Figure 1 (P = 0.000). At the fourth week, pain was 1.000 ± 0.00 for the test group, and 1.973±0.164 for the control group and their difference was significant (P = 0.000) (Figure 2). 

There were significant differences between the two groups regarding return time to work, observed infections, and body mass index (Table 1). There were no meaningful differences between groups regarding wound depth, and healing time (Figure 2). Only one case of anemia was seen in the test group. Also there was no significant difference in the groups regarding smoking (P = 0.129) (Table 1). 

## 5. Discussion

In the present study, it was found that pain in the test group was significantly lower than the control group in the first and fourth weeks. There were no significant differences between the groups regarding age and sex, while the difference in smoking, return time to work, infection, and BMI was significant.

Spyridakis et al. have investigated the effect of applying PRP on healing of the pilonidal wound. In their study, 52 patients with pilonidal sinus were subjected to surgical excision. For 22 of these cases, secondary wound closure was performed, while the 30 other cases received PRP by injection. The corresponding results were compared regarding healing time, possible complications, and return time to work. Based on the obtained results, it was found that the wound healing in the patients who received PRP was faster than the patients in the control group. The healing time in the test group was 24 days, while it was more than 30 days in the control group. Also the patients in the test group could return to work after 25 days, whereas this time was 37 days in the control group (14).

The findings in the present study are in good agreement with the previous results and the wound healing time was shorter in the test group but the difference was not meaningful. Return time to work and social activities in the test group was significantly shorter than the control group.

The findings of the Driver study were statistically significant. Therefore they concluded that diabetic foot ulcers would heal faster by treating with autologous platelet rich plasma gel (15).

The healing time of Achilles tendon was found to be shorter in Gaweda’s study and pain in the test group was reduced (16), confirming the obtained results in the present study.

Moreover, Mirsha’s study has shown that applying PRP can significantly reduce both healing time and pain in a damaged elbow tendon (17).

The corresponding results from the Tavassoli study showed that the Limberg flap has similar complications as the primary repair method, but earlier return to work and less hospital stay, lower pain score and higher comfort and satisfaction were the advantages of the Limberg flap method (18).

There are some other studies (16, 19), which confirm the present findings. The only observed disadvantage of the proposed strategy is greater possibility of infections, which may be attributed to the fact that PRP (mainly originates from blood and plasma) provides good conditions for bacteria growth. This can be overcome with increasing the dressing change times and better hygiene.
